# Dataset on extreme thunderstorm ground enhancements registered on Aragats in 2023

**DOI:** 10.1016/j.dib.2024.110554

**Published:** 2024-05-25

**Authors:** A. Chilingarian, T. Karapetyan, B. Sargsyan, D. Aslanyan, S. Chilingaryan

**Affiliations:** a Alikhanyan National Laboratory (Yerevan Physics Institute), Yerevan 0036, Armenia; b Karlsruhe Institute of Technology, Hermann-von-Helmholtz-Platz, 176344 Eggenstein-Leopoldshafen, Germany

**Keywords:** Electron accelerator, Atmospheric electric field, Natural radiation, Electron and gamma-ray energy spectra, Data mining, Correlation analysis

## Abstract

To advance high-energy atmospheric physics, studying atmospheric electric fields (AEF) and cosmic ray fluxes as an interconnected system is crucial. At Mt. Argats, simultaneous measurements of particle fluxes, electric fields, weather conditions, and lightning locations have significantly enhanced the validation of models that describe the charge structures of thunderclouds and the mechanics of internal electron accelerators. In 2023, observations of the five largest thunderstorm ground enhancements (TGEs) revealed electric fields exceeding 2.0 kV/cm at elevations just tens of meters above ground—potentially hazardous to rockets and aircraft during launch and charging operations. Utilizing simple yet effective monitoring equipment developed at Aragats, we can mitigate the risks posed by these high-intensity fields. The Mendeley dataset, comprising various measured parameters during thunderstorm activities, enables researchers to perform advanced correlation analysis and uncover complex relationships between these atmospheric phenomena. This study underscores the critical importance of integrated atmospheric studies for ensuring the safety of high-altitude operations and advancing atmospheric science.

Specification Table

**Table:** 

Subject	Earth-Surface Processes, Radiation, Data Mining and Statistical Analysis
Specific subject area	The advent of big data in scientific research has revolutionized our approach to studying high-energy phenomena in space and the terrestrial atmosphere. Data mining, visualization, and statistical analysis of the vast data sets generated by modern observatories are now indispensable tools in this domain. These observatories offer multivariate analyses of fields, radiation, and particle fluxes, providing unprecedented insights into the dynamics thunderstorms, including those with the potential for catastrophic impact. For complicated multivariate data research, it is imperative to arm researchers with robust multivariate visualization platforms enhanced with basic statistical analysis tools—such as histograms, moments, and correlations. Such a data exploration system is not just an add-on but a necessity for navigating the complexities of “new physics”.
Type of data	The dataset is organized as a table with 13 columns, the content of which can be filtered.
Data collection	The Mendeley dataset includes extensive information on Thunderstorm Ground Enhancements (TGEs, [1]) observed at the Aragats research station in 2023. The data set includes TGE maximum amplitude (maximum flux enhancement) measured by three independent particle detectors, TGE start and duration time, the electric field strength time, and distance to the lightning flash. Also, estimates of the height of the cloud base are included.
Data source location	Aragats research station of Yerevan Physics institute, Armenia. 40.25N, 44.15E, altitude 3200 m. [2]
Data accessibility	Mendeley Data [3], V1, doi: 10.17632/z4ry54hccb.1https://data.mendeley.com/datasets/z4ry54hccb/1 The Cosmic Ray Division's (CRD) databases house 20-years of multivariate measurements of particle fluxes, electric and geomagnetic fields, lightning location, and weather parameters. ADEI database [4] allowing initial data downloading in graphical and numerical form are provided. We also post several specific Mendeley datasets allowing users to make specific analysis.
Related research article	

## 1 Value of the Data

• The presented dataset disclosed the origin of high-energy cosmic rays in the atmosphere. Observed in 2023, the most intense TGEs demonstrate that cosmic ray flux amplifies tenfold within minutes and sustains elevated levels for several hours when engaging with atmospheric electric fields. Despite their critical role, the processes that modulate elementary particles within thunderclouds are not yet fully understood.
• We identified and investigated the formation and operation of an electron accelerator in thunderclouds. We demonstrated the minute-stable operation of the electron accelerator, which provides a stable electron and gamma-ray flux. We use several independent particle detectors to monitor particle fluxes. Multivariate data on particle fluxes and correspondent electric field and weather conditions opens new avenues for understanding the complex interactions between cosmic particles, electric fields, and terrestrial weather phenomena.
• In 2023, observations of the five largest thunderstorm ground enhancements (TGEs) revealed electric fields exceeding 2.0 kV/cm at elevations just tens of meters above ground—potentially hazardous to rockets and aircraft during launch and charging operations. Utilizing simple yet effective monitoring equipment developed at Aragats, we can mitigate the risks posed by these high-intensity fields. The Mendeley dataset, comprising various measured parameters during thunderstorm activities, enables researchers to perform advanced correlation analysis and uncover complex relationships between these atmospheric phenomena. This study underscores the critical importance of integrated atmospheric studies for ensuring the safety of high-altitude operations and advancing atmospheric science.
• We have compiled various aspects of TGE physics in Mendeley datasets. For example, in reference [5], we have identified TGEs suddenly interrupted by a lightning strike. This dataset includes TGEs abruptly terminated by lightning discharges, indicating their association with strong atmospheric electric fields. The dataset consists of 165 events of TGE termination by lightning observed during the period of 2012–2021 at an altitude of 3200 m above sea level on Mount Aragats in Armenia. The presented dataset includes extreme thunderstorm ground enhancements recorded on Aragats in 2023. In [6], we have documented mysterious violet atmospheric glows accompanied by intense electron flux. In [7], we have analyzed TGEs that have enabled us to recover the electron energy spectrum.

## 2 Background

In 2023, the research team on Mount Aragats in Armenia detected exceptionally large surges in cosmic rays linked to thunderstorms, with numbers rising to ten times more than on a clear day. This boost is due to strong electric fields in the clouds that act like natural accelerators, speeding up electrons to create Relativistic Runaway Electron Avalanches (RREAs, [8]). At very specific weather circumstances, these avalanches reach the Earth's surface and generate TGEs measured by facilities of the Aragats research station at an altitude of 3200 m.

Information in the data set revealed a clear pattern in when TGEs occur, shedding light on the conditions for their formation. Surprisingly, these thunderstorm-related electric fields can be incredibly strong, even at heights as low as 50 m above the ground. This observation and the consistent power of these natural electric field accelerators during thunderstorms unveil a new level of structured behavior in atmospheric electric fields that we had not recognized before.

Despite the chaotic nature of atmospheric electric fields, electron accelerators within thunderclouds demonstrated remarkable stability, maintaining a steady flux for durations of 0.5–2 min. The relative error of TGE flux at these minutes was lower than that associated with the ambient cosmic ray population, indicating a high level of stability of the electron accelerator. This finding indicates a level of organization within the atmospheric electric fields that was previously unappreciated.

Interestingly, during TGEs, there was a marked suppression of lightning activity. Lightning flashes that terminated TGEs were typically within a 10 km radius. When lightning occurred farther than 10 km, the TGEs had a prolonged duration and concluded smoothly. These observations support the hypothesis that RREAs may be precursors to lightning flashes by developing ionization channels for lightning leaders. These observations contribute to our understanding of the elusive mechanisms behind lightning initiation.

## 3 Data Description

This dataset presents the 2023 catalog of TGEs comprising measurements of various particle fluxes, environmental parameters, and near-surface electric fields on the slopes of Mt. Aragats in Armenia. The Mendeley dataset shows the EXEL file with 14 parameters of 56 TGEs observed in 2023. All multivariate measurements from the hundreds of registration channels are available from the links to each TGE event. The survey of TGE physics and TGE selection procedures can be found in the paper attached to the dataset, the details of Aragats station facilities, and data analysis methods in the WiKI section of ADEI, accessible from links in the dataset.

In the first column of the Mendeley data set (EXEL Table), we put the date of the TGE. In the second - the links to the TGE picture at the ADEI data analysis platform. The whole database with hundreds of measuring channels operated from 2008 to 2013 is available from these links. In the third to fifth columns of the table, we show the TGE significance - the percent of flux enhancement relative to fair weather value measured just before TGE. Three particle detectors were used: plastic scintillators with 1 m^2^ area and thicknesses of 1, 3, and 5 cm. We post the outside temperature and the dew point in the sixth and seventh columns. In the eighth column, we show the distance to the cloud base in meters. In the ninth column, we show the strength of the near-surface electric field (NSEF) measured on the maximum of the TGE by the EFM-100 sensor of BOLTEK company. We post the minimum and maximum NSEF values measured during TGE in the tenth column. In the eleventh column, we show the distance to the nearest lightning flash during TGE. In the twelfth column, we put the code 1 if a lightning flash abruptly terminated TGE and 0 if it didn't. In the 13th column is the time of the TGE start, and in the 14th is - the TGE duration.

## 4 Experimental Design, Materials and Methods

**Fig. 1:**
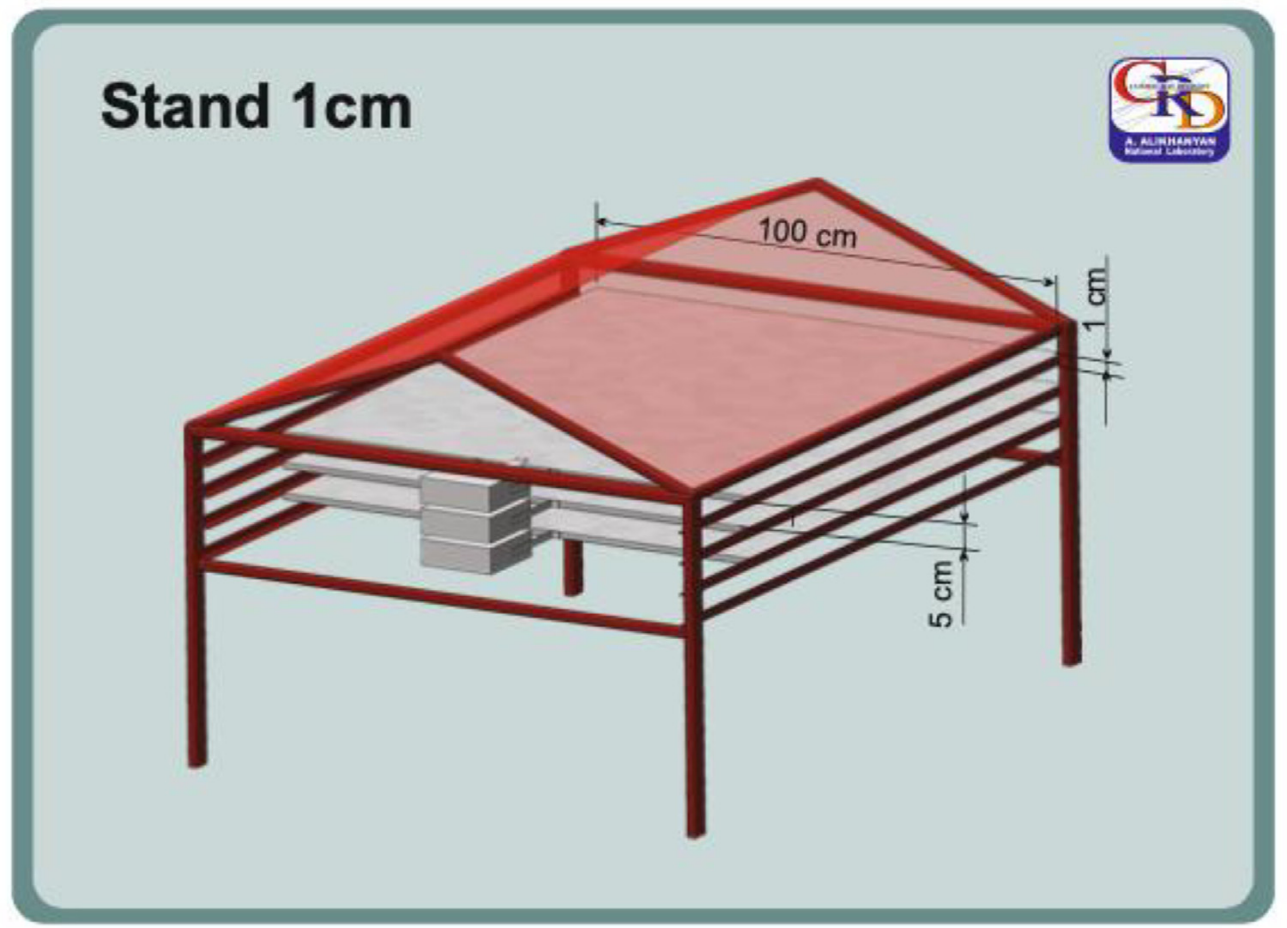
STAND1 detector setup.

The TGE detection system includes numerous plastic scintillators with an area of 1 m^2^ and 4 m^2^ and thicknesses of 1, 3, 5, 20, and 60 cm. The 1, and 3-cm thick scintillators were commissioned by the High-Energy Physics Institute in Protvino [9]. Twelve of these scintillators are part of the STAND1 network, located across the Aragats station, covering an area of ≈50,000 m^2^. Three identical units of the STAND1 network are located near three main experimental halls on Aragats. 1-cm thick scintillators are stacked vertically, see Fig. 1, and one 3-cm thick plastic scintillator stands apart. STAND1 detector has been in operation for ten years, registering spatial distribution of more than 300 TGE at millisecond time scales. The light from the scintillator through optical spectrum-shifter fibers is passed to the photomultiplier FEU-115M. The maximum luminescence is emitted at the 420-nm wavelength, with a luminescence time of about 2.3 ns. The STAND1 detector is tuned by changing the high voltage applied to the PMT and setting the thresholds for the discriminator shaper. The discrimination level is chosen to guarantee high signal detection efficiency and maximal suppression of photomultiplier noise. The efficiency of scintillators reaches 90 % and more for electron energies above 10 MeV and 2 % for gamma rays with energies above 2 MeV (for the upper scintillators). The energy threshold of the upper scintillators is 0.7–0.8 MeV, and dead time is ∼ 0.7 µs. A 50 µs time series of STAND1 detectors, synchronized with NSEF measurements and meteorological parameters, are transferred to the database. The significance of the TGE is measured by the percentage of particle flux enhancement over the background registered by the “upper” 1 cm scintillator.

**Fig. 2:**
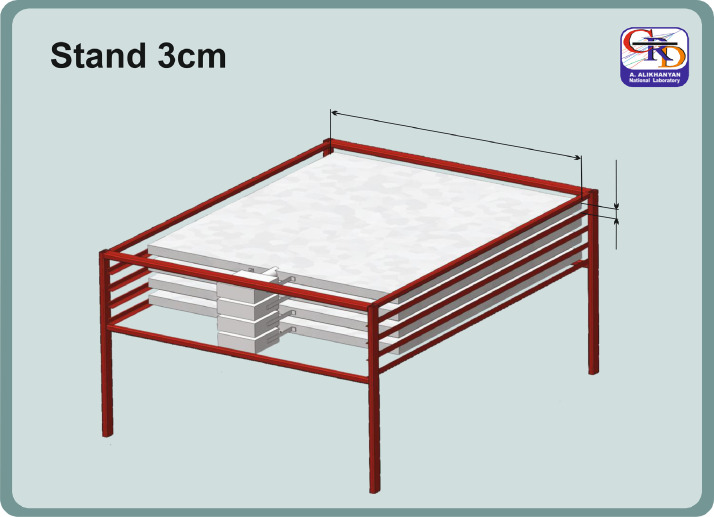
STAND3 detector setup.

The “STAND3” detector [10] comprises four layers of 3-cm-thick, 1-m^2^ sensitive area scintillators stacked vertically, using the same PMT and electronics as STAND1 detectors, see Fig. 2. In addition, DAQ electronics register and store coincidences of the signals, and 1-min histograms of energy releases for further recovering of the energy spectra. If we denote by “1” the signal from a scintillator and by “0” the absence of a signal, then the following combinations of the 4-layered detector output are stored: “1000”, most probable electron energy 10–20 MeV; “1100”— most probable electron energy 20–30 MeV; “1110”—most probable electron energy 30–40 MeV; “1111”— above 40 MeV. The energy threshold of the upper scintillator of the STAND1 detector is ∼ 5 MeV.

**Fig. 3:**
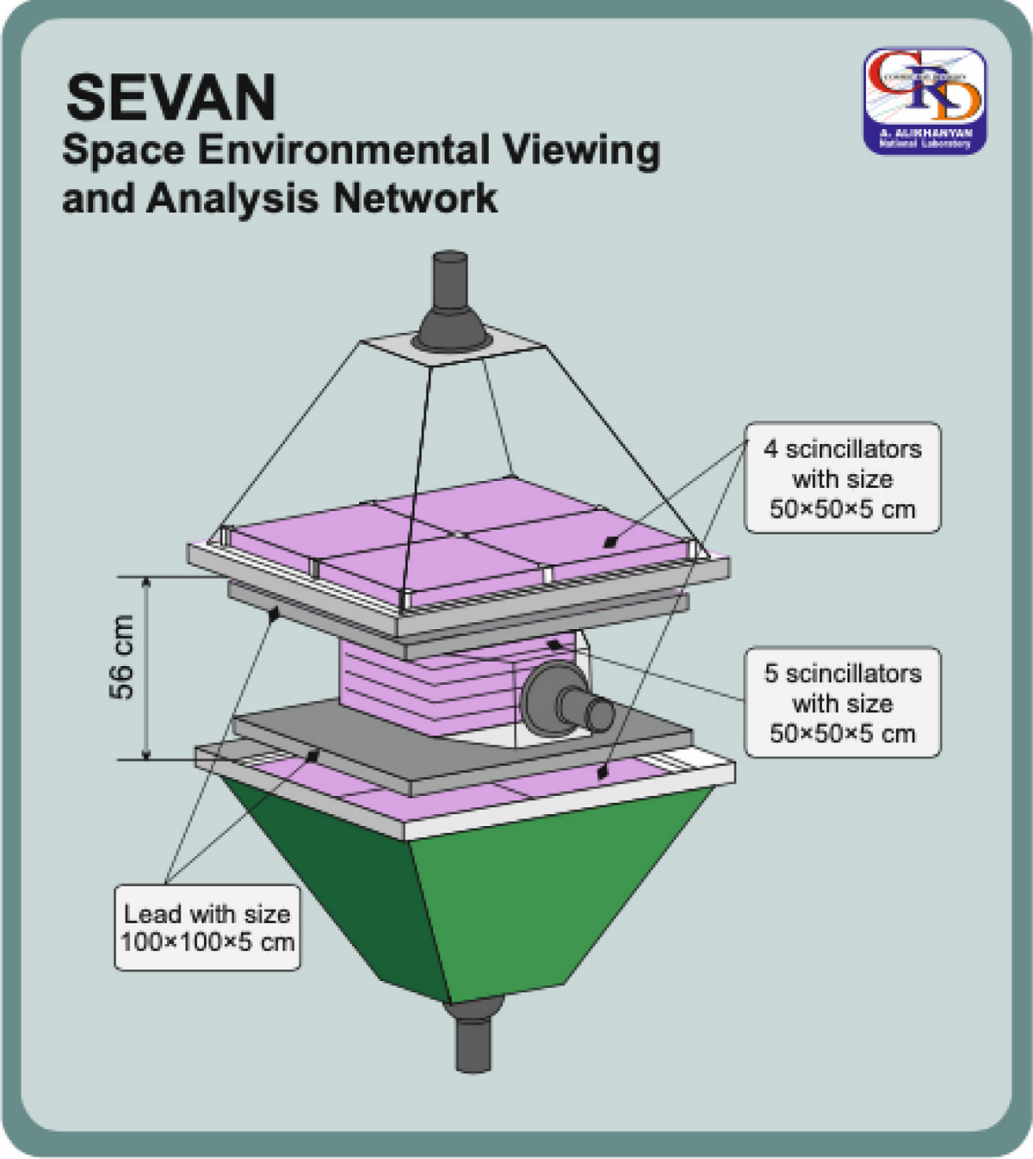
SEVAN detector setup.

SEVAN detector [11] is assembled from plastic scintillator slabs 50 × 50 × 5 cm^3^. Two identical assemblies of 100 × 100 × 5 cm^3^ scintillators (four standard slabs), two 100 × 100 × 4.5 cm^3^ lead absorbers, and a 50 × 50 × 20 cm^3^ scintillator stack (five standard slabs) are located between two identical assemblies of 100 × 100 × 5 cm^3^ scintillators (four standard slabs). Incoming neutral particles undergo nuclear reactions in the thick 20 cm plastic scintillator, producing charged particles. These particles are counted, and their energy releases are stored. In the upper 5 cm thick scintillator, charged particles are registered very effectively; however, there is not enough matter for the nuclear interactions of neutral particles. When a neutral particle traverses the top thin (5 cm) scintillator, usually no signal is produced. The absence of the signal in the upper scintillators, coinciding with the signal in the middle scintillator, indicates neutral particle traversal (gamma-ray or neutron). The coincidence of signals from the top and bottom scintillators indicates the traversal of high-energy muons. Microcontroller-based Data Acquisition (DAQ) electronics register and store all logical combinations of the detector signals for further offline analysis and online alerts issuing. If we denote by ‘‘1” the signal from a scintillator and by ‘‘0” the absence of a signal, then the following combinations of the detector output are possible: 111 and 101—traversal of high energy muon; 010— traversal of a neutral particle; 100—traversal of a low energy charged particle stopped in the scintillator or the first lead absorber. 110—traversal of a high energy charged particle stopped in the second lead absorber. 001—registration of inclined charged particles. The DAQ electronics also allow the remote control of the PMT high voltage and other important parameters of the detector. The energy threshold of the upper scintillator of the SEVAN detector is ∼10 MeV (Fig. 3).

The outside temperature during the TGE, the dew point temperature, and an estimate of the cloud base height are obtained with the Davis vantage pro plus weather station with a frequency of measurements of one per minute [12]. The relative to instrument elevation (3200 m) cloud base height was calculated from the difference between the outside temperature and dew point measurements [13]:

$$h\;\left(m\right)\approx \;122\;*\;\left(temp\;-\;dew\;point\right)C^{\circ }$$

This method is most effective and accurate for convective clouds such as Cumulus, towering Cumulus, and Cumulonimbus.

Boltek's EFM-100 electric field monitor ([14], frequency 20 Hz) measures NSEF. Lightning is detected as an abrupt (50–500 ms duration) change in the static electric field (with amplitude up to ≈ 40 kV/m).

## Ethics Statement

The authors have read and followed the ethical requirements for publication in Data in Brief and confirm that the current work does not involve human subjects, animal experiments, or any data collected from social media platforms.

## CRediT authorship contribution statement

**A. Chilingarian:** Conceptualization, Supervision, Writing – original draft. **T. Karapetyan:** Data curation. **B. Sargsyan:** Visualization, Investigation. **D. Aslanyan:** Visualization, Software. **S. Chilingaryan:** Methodology, Software.

## Data Availability

Extreme thunderstorm ground enhancements registered on Aragats in 2023 (Original data) (Mendeley Data).
